# A cross-sectional study exploring the relationship between burnout, absenteeism, and job performance among American nurses

**DOI:** 10.1186/s12912-019-0382-7

**Published:** 2019-11-21

**Authors:** Liselotte N. Dyrbye, Tait D. Shanafelt, Pamela O. Johnson, Le Ann Johnson, Daniel Satele, Colin P. West

**Affiliations:** 1 Mayo Clinic Program on Physician Well-Being, Mayo Clinic, 200 First Street SW, Rochester, MN 55905 USA; 2 Stanford School of Medicine, Stanford, CA USA; 3 Department of Nursing, Mayo Clinic, Rochester, MN USA; 4 Department of Health Sciences Research, Mayo Clinic, Rochester, MN USA

**Keywords:** Nurses, Burnout, psychological, Presenteeism, Absenteeism, Job performance

## Abstract

**Background:**

Studies suggest a high prevalence of burnout among nurses. The aim of this study was to evaluate the relationship between burnout among nurses and absenteeism and work performance.

**Methods:**

A national sample of U.S. nurses was sent an anonymous, cross-sectional survey in 2016. The survey included items about demographics, fatigue, and validated instruments to measure burnout, absenteeism, and poor work performance in the last month.

**Results:**

Of the 3098 nurses who received the survey, 812 (26.2%) responded. The mean age was 52.3 years (SD 12.5), nearly all were women (94.5%) and most were married (61.9%) and had a child (75.2%). Participating nurses had a mean of 25.7 (SD 13.9) years of experience working as nurse and most held a baccalaureate (38.2%) or masters of science (37.1%) degree in nursing. A quarter worked in the inpatient setting (25.5%) and the average hours worked per week was 41.3 (SD 14.1). Overall, 35.3% had symptoms of burnout, 30.7% had symptoms of depression, 8.3% had been absent 1 or more days in the last month due to personal health, and 43.8% had poor work performance in the last month. Nurses who had burnout were more likely to have been absent 1 or more days in the last month (OR 1.85, 95% CI 1.25–2.72) and have poor work performance (referent: high performer; medium performer, OR 2.68,95% CI 1.82–3.99; poor performer, OR 5.01, 95% CI 3.09–8.14). After adjusting for age, sex, relationship and parental status, highest academic degree, practice setting, burnout, depression, and satisfaction with work-life integration, nurses who were more fatigued (for each point worsening, OR 1.22, 95% CI 1.10–1.37) were more likely to have had absenteeism while those who worked more hours (for each additional hour OR 0.98, 95% CI 0.96–1.00) were less likely to have had absenteeism. Factors independently associated with poor work performance included burnout (OR 2.15, 95% CI 1.43–3.24) and fatigue (for each point of worsening, OR 1.22, 95% CI 1.12–1.33).

**Conclusions:**

These findings suggest burnout is prevalent among nurses and likely impacts work performance.

## Background

Professional burnout [1] is alarmingly prevalent among U.S. nurses with studies reporting rates of 35–45% [2–7]. Burnout is a syndrome characterized by feelings of energy exhaustion, cynicism related to one’s job, and reduced professional efficacy that stems from chronic work-related stress [8]. Excessive workload, inadequate staffing, values conflicts, inadequate rewards, and poor work environment (e.g., insufficient autonomy, lack of administrative support, poor physician-nurse relationships) increase the risk of burnout among nurses [3, 4, 9–17]. Studies suggest the consequences of burnout among nurses include lower willingness to lead, suboptimal quality of patient care, lower inpatient satisfaction ratings, more health care-associated infections, and increased patient mortality ratios [3, 4, 18–21].

Previous studies also suggest nurses with burnout are more likely to be dissatisfied with their job and intend to or actually leave their place of employment [3, 22–28]. Few studies, however, have examined the potential impact of nurse burnout on absenteeism and work performance [29]. Systematic reviews of articles published between 1950 and 2016 on absenteeism and presenteeism (impaired performance at work) [30] and between 1986 and 2006 on absenteeism [31] in nurses identified only two studies examining the relationship between burnout and presenteeism. One study of 73 registered nurses reported that higher levels of burnout were associated with worse supervisor rated job performance and more self-reported absences [32]. In the second study of 258 nurses working in the Netherlands, a bi-directional relationship was found between burnout and presenteeism [33]. We identified another study of 404 nurses working in an institution for people with learning difficulties where the emotional exhaustion domain of burnout was associated with higher self-reported absenteeism [34], and in a 1989 study of 106 nurses working in long-stay settings, emotional exhaustion predicted absences in the subsequent 12 months [35]. Important limitations of previous studies, however, include being conducted more than a decade ago or outside the U.S., having small sample sizes of nurses from a single specialty or practice setting, using only the emotional exhaustion domain of burnout, or being unable to account for potential confounding factors such as mood disorders and fatigue [32–35].

To further our knowledge about the relationship between burnout and self-reported absenteeism and job performance among nurses, we conducted a national survey of U.S. nurses using validated measures. We hypothesized that nurses who had burnout would be more likely to report absenteeism and lower job performance than nurses without burnout.

## Methods

We adhered to Strengthening the Reporting of Observational Studies in Epidemiology (STROBE) guidelines and methodology.

### Participants

In November 2016 we conducted a cross-sectional exploratory study [2]. obtained a random sample of 3150 U.S. registered nurses’ provided by Redi-Data, a company that maintains over 5.8 million postal addresses and over 1.8 million e-mail addresses for U.S. nurses obtained from state licensing data (more information available: http://www.redidata.com/healthcare-lists/mailing-email-lists/state-licensed-nurses-rns-mailing-email-lists). There were 3 duplicates, resulting in emails being sent to 3147 nurses. The e-mail informed the nurses of the purpose of the study (e.g., to better understand the factors that contribute to satisfaction among U.S. nurses) and provided a link to the survey. Non-responders to the web-survey received a paper survey in the mail. From the sample of 3147 nurses, we were unable to reach 47 (no functional e-mail or address) and were notified 2 were deceased, resulting in 3098 nurses having received an invitation to participate in the study. Participation was voluntary and all responses were anonymous. Nurses who indicated they had an associate degree or higher (e.g., baccalaureate degree in nursing, masters of science in nursing, doctorate of nursing practice, or doctorate of nursing) and were not advance practice providers (i.e., certified nurse practitioners, certified registered nurse anesthetist, certified clinical nurse specialists, certified nurse midwife) were included in this analysis. We excluded advance practice providers as contributors and consequences of their work stress likely vary from other nurses given their broader scope of practice.

### Study measures

The survey items can be found in the Additional file 1. Items on the survey inquired about personal characteristics and professional characteristics. The survey included questions about demographics (age, gender, relationship status [single, married, partnered, widowed], parental status [yes/no]), practice characteristics (work hours, current practice setting, years working as a nurse, highest academic degree related to nursing, advanced practice certification), satisfaction with work-life balance, and standardized instruments to measure absenteeism, work performance, burnout, depression, and fatigue.

To measure absenteeism (i.e., work days missed due to mental or physical illness) and self-rated work performance we used the World Health Organization Health and Work Performance Questionnaire (HPQ), an instrument used by the WHO in 25 countries, that has excellent reliability and validity, and has been validated in multiple occupation samples in the U.S. and abroad and in samples of individuals employed in the health care sector [36–40]. Data obtained from this instrument on self-reported absenteeism and work performance has good concordance with employee archival measures of absenteeism, daily diary reports, and worker performance in a variety of professions [36–38, 41]. For absenteeism, respondents were asked to indicate the number of entire work days they missed due to personal physical or mental health problems in the last month. In samples of U.S. workers, good concordance has been found between HPQ self-reported absenteeism and employer payroll records in multiple occupations (Pearson correlations of 0.66 to 0.71 for 28 day recall) [37, 38]. We dichotomized responses into those who had been absent one or more days due to a personal health problem in the last month versus those who had not.

For work performance, the HPQ has a series of three questions where the respondent uses a 0 (worse performance) to 10 (top performance) scale to rate their own work performance. First, respondents are asked to rate the usual performance of most workers in a similar job to their own. Then, they are asked to rate their own usual job performance over the past year or two. Lastly, the respondent is asked to rate their own overall job performance on the days they worked during the past 4 weeks. These questions are general so that they apply to all occupations, but focused enough to allow for individual reflection. The first and second questions are for memory priming only, and response to the third question is used for analysis. The lower end of the scale is truncated at 0–7 as only a small percentage of respondents rate themselves less than 7 [37, 38].

We categorized respondents into low performers (self-ratings of 7 or lower), medium performers (self-ratings of 8) and high performers (self-ratings of 9 or higher) as previous studies of U.S. workers have reported that individuals who rate themselves 7 or lower have statistically significantly lower supervisor work performance ratings than do individuals with self-ratings of 8, and that individuals who rate themselves at an ‘8’ have significantly worse supervisor work performance ratings than individuals with self-ratings of 9 and above [37, 38, 42]. For example, in a study of reservation agents, in comparison to individuals with a HPQ work performance rating of 9 or higher, those with HPQ work performance ratings of 7 or lower had 3.2-times greater odds of poor supervisor ratings and individuals with a HPQ work performance rating of 8 had a 2.4-times greater odds of poor supervisor ratings [38]. We further dichotomized individuals as having poor work performance or not based on if their self-rating score was less than or equal to 8 or not.

Previous validation studies in US workers have demonstrated significant associations between HPQ scores and payroll records and job performance assessments by supervisors and other records (receiver operating characteristic curves of 0.58–0.72 in US workers) [37, 38]. The HPQ has been used widely in samples of workers [39, 40, 43], although not specifically in nurses.

We used the full 22-item Maslach Burnout Inventory (MBI) Human Services Survey to measure burnout [44]. The MBI includes three subscales: emotional exhaustion, depersonalization, and low sense of personal accomplishment. Individuals are asked to indicate how often they have experience various job-related feelings (response options: never, a few times a year or less, once a month or less, a few times a month, once a week, a few times a week, every day). Psychometric properties of the MBI (i.e., reliability coefficients, test re-test reliability, convergent validity, and discriminant validity) among human service professionals can be found in the manual [1] and has recently been summarized [45]. Previous studies showing relationships between burnout, as measured by the MBI, and health care outcomes provide additional validity data [3, 46]. Consistent with other studies, nurses were considered to have symptoms of burnout if they scored high on the emotional exhaustion (score ≥ 27) and/or depersonalization (score ≥ 10) subscale [47, 48].

We identified symptoms of depression by using the 2-item Primary Care Evaluation of Mental Disorders (PRIME MD) [49], a screening tool that performs as well as longer instruments [50]. The PRIME MD inquiries about symptoms over the past month and has a sensitivity of 86 to 96% and a specificity of 57 to 75% for major depressive disorder [49, 50]. Similar to the approach described by West et al. [51], we assessed fatigue on a standardized linear analog scale (0 = “As bad as it can be”; 10 = “As good as it can be”) where lower score indicates a greater degree of fatigue [52]. Standardized linear analog scales have been widely validated across medical conditions and populations [53–57].

### Statistical analysis

We calculated standard descriptive statistics. Associations between variables were evaluated using Fisher exact or chi-square tests, as appropriate. We conducted multivariable analysis (forward stepping logistic regression with backwards stepping confirmation) to identify personal and professional characteristics independently associated with the dependent variables absenteeism (1 or more work days missed due to personal mental or physical health) and self-rated poor work performance (HPQ self-rated job performance of 8 or below). Variables included in the multivariable models were: relationship [not dichotomized] and parental status, work hours in the past 7 days, academic degree, practice setting, burnout, depression, fatigue, and satisfaction with work-life integration. Age and sex were kept in the models because are traditional confounders; burnout was also kept in all models. All variables entered into the models were chosen a priori. We used a 5% type I error rate and a two-sided alternative. All analysis was conducted using SAS version 9 (SAS Institute, Cary, NC).

## Results

### Demographic and descriptive results

Of the 3098 nurses who received the survey, 812 (26.2%) responded [2]. Among the responders, 175 were advanced practice nurses and were excluded from this analysis, resulting in a final sample of 637 nurses. The demographics and professional characteristics of the 637 participating nurses are summarized in Table 1. The mean age was 52.3 years (standard deviation, SD 12.5), nearly all were women (94.5%) and most were married (61.9%) and had a child (75.2%). Participating nurses had a mean of 25.7 (SD 13.9) years of experience working as nurse and most held a baccalaureate (38.2%) or masters of science (37.1%) degree in nursing. A quarter worked in the inpatient setting (25.5%) and the average hours worked per week was 41.3 (SD 14.1).

**Table 1:** Personal and Professional Characteristics of the 637 Participating Nurses

Nurses	
Female sex, No. (%)	596 (94.5%)
Age, Mean (SD)	52.3 (12.5)
Relationship status, No. (%)	
Single	169 (26.8%)
Married	390 (61.9%)
Partnered	43 (6.8%)
Widowed	28 (4.4%)
Missing	7
Have children, No. (%)	475 (75.2%)
Highest earned academic degree in or related to nursing, No. (%)	
Associate degree	67 (10.9%)
Baccalaureate degree in nursing	235 (38.2%)
Masters of science in nursing	228 (37.1%)
Doctorate of Nursing Practice or Nursing (PhD)	28 (4.5%)
Other	57 (9.3%)
Hours worked past week, mean (SD)	41.3 (14.1)
Years of experience working in nursing, mean (SD)	25.7 (13.9)
Current practice setting, No. (%)	
Inpatient	153(25.6%)
Outpatient	129 (21.6%)
Community-based public health [1]	60 (10.0%)
Non-clinical, such as management	49 (8.2%)
Other	207 (34.6%)
Missing	39

^1^Includes hospice, home health, and public health

The mean emotional exhaustion score was 21.2 (*N* = 617/637, SD 12.3) with 30.5% (188/617) having high emotional exhaustion. The mean depersonalization score was 5.4 (*N* = 609/637, SD 5.3) with 20.0% (122/609) having high depersonalization. The mean personal accomplishment score was 39.1 (*N* = 609/637, SD 6.8) with 19.0% (116/609) having low personal accomplishment. Overall, 35.3% (218/617) had at least one symptom of burnout. Nearly a third (192/625, 30.7%) had symptoms of depression. The mean fatigue score was 6.0 (*N* = 608/637, SD 2.4). Nearly 60% felt that their work schedule left enough time for personal/family life.

Absenteeism was reported by 16.6% with half of this group having missed 1 day in the past month due to a personal health problem and the other half missing more than 1 day. Most (56.2%) nurses rated themselves as a high work performer (score of 9 or higher). Slightly more than a quarter (28.2%) of nurses rated themselves as a medium work performer (score of 8), and 15.6% rated themselves as a poor work performer (score of 7 or below).

### Associations with burnout

**Fig. 1:**
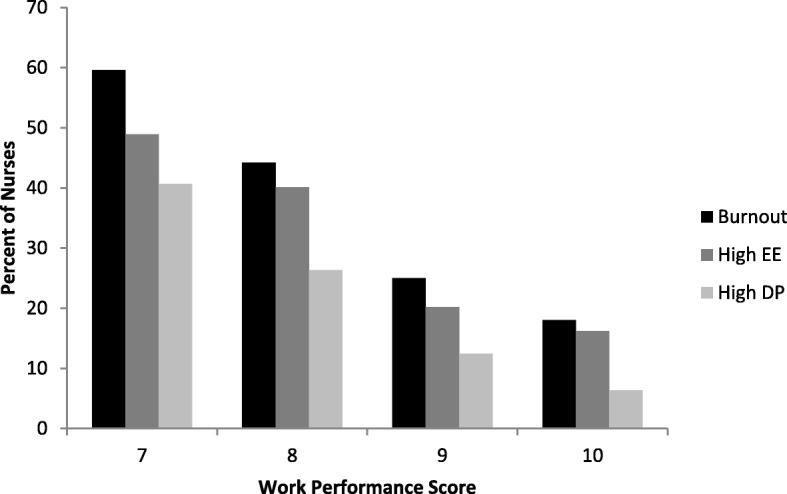
Relationship between burnout and work performance

In univariate analysis (Table 2) nurses who had burnout were more likely to have been absent 1 or more days in the last month (odds ratio [OR] 1.85, 95% confidence interval [CI] 1.25–2.72). Nurses with burnout were also more likely to rate their own job performance as worse (referent: high performer [scores of 9 and above]; medium performer [scores of 8], OR 2.68, 95% CI 1.82–3.99; poor performer [scores of 7 or lower] OR 5.01, 95% CI 3.09–8.14). Fig. 1 shows the relationship between burnout and work performance. As work performance increased, the prevalence of overall burnout, high emotional exhaustion, and high depersonalization decreased.

**Table 2:** Absenteeism and Work Performance among Nurses with and without Burnout

	Burnout *N* = 218	No Burnout *N* = 399	Unadjusted odds ratio (95% CI)^1^
Absenteeism due to person health in last month, No. (%)			
0 days	155 (78.7%)	327 (85.8%)	reference
≥1 days	42 (21.3%)	54 (14.2%)	1.85 (1.25–2.72)
Work performance in the last month,^a^ No. (%)			
High performer	77 (36.8%)	262 (66.2%)	reference
Medium performer	76 (36.4%)	96 (24.2%)	2.69 (1.82–3.99)
Poor performer	56 (26.8%)	38 (9.6%)	5.01 (3.09–8.14)

^a^ Based on work performance score on the World Health Organization Health and Work Performance Questionnaire. Individuals with self-ratings of 9 and above are considered ‘high performers, self-ratings of 8 are considered ‘medium performers,’ and self-ratings of 7 or lower are considered ‘low performers’

### Multivariable analysis

Next, we performed multivariable analysis to identify personal and professional characteristics independently associated with absenteeism (one or more days in the past month) and poor work performance (Table 3). After controlling for age, sex, and burnout, nurses who were more fatigued (for each point worsening, OR 1.22, 95% CI 1.10–1.37) were more likely to have had absenteeism while those who worked more hours (for each additional hour OR 0.98, 95% CI 0.96–1.00) were less likely to have had absenteeism. Lastly, after controlling for sex, burnout (OR 2.15, 95% CI 1.43–3.24), fatigue (for each point of worsening OR 1.22, 95% CI 1.12–1.33) and being older (for each year older, OR 0.97, 95% CI 0.95–0.98) were independently associated with higher odds of low work performance.

**Table 3:** Multivariate Analysis to Identify Factors Independently Associated with Absenteeism and Poor Work Performance^a^

Predictor	OR (95% CI)	*P*-value
Absenteeism^b^		
Burned out (vs. not)	1.03 (0.61–1.74)	0.91
Fatigue (for each point worsening)	1.22 (1.10–1.37)	< 0.001
Age (each year older)	0.99 (0.97–1.01)	0.44
Female (vs Male)	2.86 (0.66–12.44)	0.16
Hours in last 7 days (for each additional hour)	0.98 (0.96–1.00)	0.05
Poor Work Performance^c^		
Burned out (vs. not)	2.15 (1.43–3.24)	0.0002
Fatigue (for each point worsening)	1.22 (1.12–1.33)	< 0.0001
Age (each year older)	0.97 (0.95–0.98)	< 0.0001
Female (vs Male)	0.68 (0.30–1.51)	0.34

^a^Factors in the model: age, relationship status, sex, have children, work hours/week, academic degree (undergraduate [associate or BA] vs. graduate [Maters, Doctorate, other]), practice setting [inpatient vs. not], burnout, depression, fatigue, satisfaction with work-life balance. Forward stepping logistic regression w/ backwards stepping confirmatory run. Age and sex were kept in the model because these are traditional confounders; burnout was also kept in the models

^b^Missed ≥ 1 day of work due to personal health in the last month

^c^Self-rated work performance score 0–8 on the World Health Organization Health and Work Performance Questionnaire

## Discussion

In this national study of U.S nurses, over a third had substantial symptoms of burnout, and, similar to the findings reported in a study conducted in the Netherlands [33], those with burnout were more likely to self-report poor work performance. We did not find a statistically significant association between burnout and absenteeism. However, absence from work due to personal illness was uncommon in this sample, and the wide confidence interval around this effect estimate [58] does not allow a clinically important association between burnout and absenteeism to be excluded. A previous study conducted in Europe suggested burnout predicts subsequent absenteeism among nurses [35]. Among non-health care employees, burnout as well as poor work performance has been shown to be a predictor of future work absences in longitudinal studies [40, 59]. In sum, these findings suggest burnout remains prevalent among nurses and likely impacts work perfromance.

Nurses in our cohort who had symptoms of burnout were also more likely to have reduced on the work performance, independent of fatigue and other factors. Poor work performance may have a greater negative impact on patient care (as the nurse is not replaced on their shift) [60] and be more costly than absenteeism [61]. A previous study of inpatient nurses in North Carolina found an association between presenteeism and patient falls and medication errors, with estimated costs of $1346 per nurse annually in North Carolina (2009), or if extrapolated to all nurses in the U.S., just under $2 billion annually [60].

In this cohort, 16% reported missing at least 1 day at work in the past month due to a personal health issue. In a study of over 6000 nurses from seven countries the reported prevalence of missing work over the past 3 months ranged from 10% (South Korea) to 74% (Iceland), and was 56% among nurses working in the US [62]. In that international study, older nurses were less likely to report absenteeism, whereas nurses who worked full-time, had overtime, and perceived staffing to be inadequate on their unit were more likely to report absenteeism after controlling for country and hospital clustering.

Findings from this study suggest organizational investment in strategies aimed at reducing burnout among nurses is needed, and if successful, likely to have a positive return on investment and benefit nursing-sensitive quality of care indicators. Such strategies should take aim at the environment that nurses work in and work-related contributors to stress [3, 9], rather than solely focus on individual strategies to deal with stress. Intervention studies with appropriate control groups are needed to inform evidence-based organizational strategies to address nurse burnout and related issues.

This study has several limitations. First, the response rate was 26.2%. Although this is typical of national surveys, our findings are vulnerable to response bias. Our responders, however, were fairly typical of US nurses with respect to age, sex, highest academic degree related to nursing, and work hours [63, 64]. Furthermore the prevalence of burnout in this cohort was similar to that found in previous studies of nurses [2, 3], suggesting our findings may be comparable. Second, we explored a limited number of personal and professional characteristics hypothesized to be associated with absenteeism and work performance. There are likely to be additional factors beyond those measured in this study that also influence these outcomes. Third, we did not collect objective data on absenteeism or work performance. We did, however, use a validated measure with demonstrated concordance with employee archival measures of absenteeism, daily diary reports, and supervisor ratings [36–38, 41].

Strengths of this study include use of the criterion standard burnout assessment instrument (the Maslach Burnout Inventory) and statistical adjustment for symptoms of depression and fatigue. Future research should explore additional factors likely to impact absenteeism and work performance, leverage employer data on absences and job performance, use longitudinal study designs, and further explore the effects of absenteeism on the colleagues impacted by the nurses who are absent [65].

## Conclusion

In conclusion, in this study of U.S. nurses we found nearly 1 in 3 had symptoms of burnout, and burnout doubled the odds of low work performance. One in six self-reported absenteeism in the last month due to a personal illness. Although we did not find a statistically significant relationship between burnout and absenteeism, one in six self-reported absenteeism in the last month due to a personal illness. To improve work performance, organizations should address work-related stressors contributing to nurse burnout and absenteeism.

## Supplementary information

**Additional file 1.** Nurse survey.

## Data Availability

Not available.

## Abbreviations

HPQ: World Health Organization Health and Work Performance Questionnaire
MBI: Maslach Burnout Inventory
PRIME MD: Primary Care Evaluation of Mental Disorders
SD: Standard deviation
OR: Odds ratio
CI: Confidence interval

## References

[1] Maslach C, Jackson SE, Leiter MP (1996). Maslach burnout inventory manual.

[2] Dyrbye LN, Johnson PO, Johnson LM, Satele D, Shanafelt TD. Efficacy of the well-being index to identify distress and well-being in US nurses. Nurs Res. 2018;67(6):447–55. 10.1097/NNR.0000000000000313 30138124

[3] Dyrbye LN, Shanafelt TD, Sinsky CA, Cipriano PF, Bhatt J, Ommaya A, West CP, Meyers D (2017). Burnout among health care professionals: A call to explore and address this underrecognized threat to safe, high-quality care.

[4] Aiken LH, Sermeus W, Van den Heede K (2012). Patient safety, satisfaction, and quality of hospital care: cross sectional surveys of nurses and patients in 12 countries in Europe and the United States. BMJ.

[5] Moss M, Good VS, Gozal D, Kleinpell R, Sessler CN (2016). An official critical care societies collaborative statement: burnout syndrome in critical care healthcare professionals: A call for action. Crit Care Med.

[6] Pradas-Hernández L, Ariza T, Gómez-Urquiza JL, Albendín-García L, De la Fuente EI. Cañadas-De la Fuente GA. Prevalence of burnout in paediatric nurses: A systematic review and meta-analysis. PLoS One. 2018;13(4):e0195039. 10.1371/journal.pone.0195039. 10.1371/journal.pone.0195039 PMC5918642 29694375

[7] Li H, Cheng B, Zhu XP (2018). Quantification of burnout in emergency nurses: A systematic review and meta-analysis. Int Emerg Nurs.

[8] International Classification of Diseases for Mortality and Morbidity Statistics. Version 4/2019. QD85 Burn-out. 2019. at https://icd.who.int/browse11/l-m/en#/http://id.who.int/icd/entity/129180281, Accessed 10/7/2019.)

[9] Woodhead EL, Northrop L, Edelstein B (2016). Stress, social support, and burnout among long-term care nursing staff. J Appl Gerontol.

[10] Pisanti R. Van der Doef mv, Maes S, Meier LL, Lazzari D, Violani C. How changes in psychosocial job characteristics impact burnout in nurses: A longitudinal analysis. Front Psychol. 2016;7:1082. Published online 2016 Jul 26. 10.3389/fpsyg.2016.01082. 10.3389/fpsyg.2016.01082 PMC4960268 27507952

[11] McHugh MD, Ma C (2014). Wage, work environment, and staffing: effects on nurse outcomes. Policy, Polit Nurs Pract.

[12] Kutney-Lee A, Wu ES, Sloane DM, Aiken LH (2013). Changes in hospital nurse work environments and nurse job outcomes: an analysis of panel data. Int J Nurs Stud.

[13] Pisanti R (2012). JobDemands-control-social SupportModel and coping strategies: predicting burnout and wellbeing in a group of ItalianNurses. Med Lav.

[14] Rushton CH, Batcheller J, Schroeder K, Donohue P (2015). Burnout and resilience among nurses practicing in high-intensity settings. Am J Crit Care.

[15] Shin S, Park JH, Bae SH (2018). Nurse staffing and nurse outcomes: A systematic review and meta-analysis. Nurs Outlook.

[16] Li B, Bruyneel L, Sermeus W (2013). Group-level impact of work environment dimensions on burnout experiences among nurses: A multivariate multilevel probit model. Int J Nurs Stud.

[17] Harris DA, Haskell J, Cooper E, Crouse N, Gardner R (2018). Estimating the association between burnout and electronic health record-related stress among advanced practice registered nurses. Appl Nurs Res.

[18] Cimiotti JP, Aiken LH, Sloane DM, Wu ES (2012). Nurse staffing, burnout, and health care-associated infection.[erratum appears in am J infect control. 2012 Sep;40(7):680]. Am J Infect Control.

[19] Welp A, Meier LL, Manser T (2015). Emotional exhaustion and workload predict clinician-rated and objective patient safety. Front Psychol.

[20] Al Sabei SD, Ross AM, Lee CS (2019). Factors influencing nurses' willingness to lead. J Nurs Manag.

[21] Galletta M, Portoghese I, D'Aloja E (2016). Relationship between job burnout, psychosocial factors and health care-associated infections in critical care units. Intensive Crit Care Nurs.

[22] Hayes LJ, O'Brien-Pallas L, Duffield C (2012). Nurse turnover: a literature review - an update. Int J Nurs Stud.

[23] Fida R, Laschinger HKS, Leiter MP (2018). The protective role of self-efficacy against workplace incivility and burnout in nursing: A time-lagged study. Health Care Manag Rev.

[24] Dutra HS, Cimiotti JP, Guirardello EDB (2018). Nurse work environment and job-related outcomes in Brazilian hospitals. Appl Nurs Res.

[25] Havaei F, Macphee M, Susan DV (2016). RNs and LPNs: emotional exhaustion and intention to leave. J Nurs Manag.

[26] Nantsupawat A, Kunaviktikul W, Nantsupawat R, Wichaikhum OA, Thienthong H, Poghosyan L (2017). Effects of nurse work environment on job dissatisfaction, burnout, intention to leave. Int Nurs Rev.

[27] Lee HF, Chiang HY, Kuo HT (2019). Relationship between authentic leadership and nurses' intent to leave: The mediating role of work environment and burnout. J Nurs Manag.

[28] Palazoglu CA, Koc Z (2019). Ethical sensitivity, burnout, and job satisfaction in emergency nurses. Nurs Ethics.

[29] AACN (2017). Fact sheet: nursing shortage. American Association of Colleges of Nursing.

[30] Brborović H, Daka Q, Dakaj K, Brborović O. Antecedents and associations of sickness presenteeism and sickness absenteeism in nurses: A systematic review. Int J Nurs Pract. 2017;23(6). 10.1111/ijn.12598. Epub 2017 Nov 1. 10.1111/ijn.12598 29094426

[31] Davey MM, Cummings G, Newburn-Cook CV, Lo EA (2009). Predictors of nurse absenteeism in hospitals: a systematic review. J Nurs Manag.

[32] Parker PA, Kulik JA (1995). Burnout, self- and supervisor-rated job performance, and absenteeism among nurses. J Behav Med.

[33] Demerouti E, Blanc PM, B. bakker A, Schaufeli W, Hox J (2009). Present but sick: A three-wave study on job demands, presenteeism and burnout. Career Dev Int.

[34] Bekker MHJ, Croon MA, Bressers B (2005). Childcare involvement, job characteristics, gender and work attitudes as predictors of emotional exhaustion and sickness absence. Work Stress.

[35] Firth H, Britton P (1989). "burnout," absence and turnover amongst British nursing staff. J Occup Psychol.

[36] AlHeresh R, LaValley MP, Coster W, Keysor JJ (2017). Construct validity and scoring methods of the World Health Organization: health and work performance questionnaire among workers with arthritis and Rheumatological conditions. J Occup Environ Med.

[37] Kessler RC, Ames M, Hymel PA (2004). Using the World Health Organization health and work performance questionnaire (HPQ) to evaluate the indirect workplace costs of illness. J Occup Environ Med.

[38] Kessler RC, Barber C, Beck A (2003). The World Health Organization health and work performance questionnaire (HPQ). J Occup Environ Med.

[39] Scuffham PA, Vecchio N, Whiteford HA (2014). Exploring the validity of HPQ-based presenteeism measures to estimate productivity losses in the health and education sectors. Med Decis Mak.

[40] Suzuki T, Miyaki K, Song Y (2015). Relationship between sickness presenteeism (WHO-HPQ) with depression and sickness absence due to mental disease in a cohort of Japanese workers. J Affect Disord.

[41] Merikangas KR, Ames M, Cui L (2007). The impact of comorbidity of mental and physical conditions on role disability in the US adult household population. Arch Gen Psychiatry.

[42] Johns G, Miraglia M (2015). The reliability, validity, and accuracy of self-reported absenteeism from work: a meta-analysis. J Occup Health Psychol.

[43] Wang PS, Simon GE, Avorn J (2007). Telephone screening, outreach, and Care Management for Depressed Workers and Impact on clinical and work productivity outcomes. JAMA.

[44] Maslach C, Jackson SE, Leiter MP (2016). Maslach Burnout Inventory.

[45] Validated Instruments to Assess Work-Related Dimensions of Well-Being. 2018. (Accessed 2018, 2018, at https://nam.edu/valid-reliable-survey-instruments-measure-burnout-well-work-related-dimensions/.)

[46] West CP, Dyrbye LN, Shanafelt TD (2018). Physician burnout: contributors, consequences and solutions. J Intern Med.

[47] Shanafelt TD, Hasan O, Dyrbye LN (2015). Changes in burnout and satisfaction with work-life balance in physicians and the general US working population between 2011 and 2014.[erratum appears in Mayo Clin Proc. 2016 Feb;91(2):276]. Mayo Clin Proc.

[48] Shanafelt TD, Boone S, Tan L (2012). Burnout and satisfaction with work-life balance among US physicians relative to the general US population. Arch Intern Med.

[49] Spitzer RL, Williams JB, Kroenke K (1994). Utility of a new procedure for diagnosing mental disorders in primary care. The PRIME-MD 1000 study. JAMA.

[50] Whooley MA, Avins AL, Miranda J, Browner WS (1997). Case-finding instruments for depression. Two questions are as good as many. J Gen Intern Med.

[51] West CP, Tan AD, Habermann TM, Sloan JA, Shanafelt TD (2009). Association of resident fatigue and distress with perceived medical errors. JAMA.

[52] Aitken RC (1969). Measurement of feelings using visual analogue scales. Proc R Soc Med.

[53] Singh JA, Satele D, Pattabasavaiah S, Buckner JC, Sloan JA (2014). Normative data and clinically significant effect sizes for single-item numerical linear analogue self-assessment (LASA) scales. Health Qual Life Outcomes.

[54] Rummans T, Clark MM, Sloan JA (2006). al. e. Impacting quality of life for patients with advanced cancer with a structured multidisciplinary intervention: a randomized controlled trial. J Clin Oncol.

[55] Sloan JA, Zhao X, Novotny PJ (2012). Relationship between deficits in overall quality of life and non-small-cell lung cancer survival. J Clin Oncol.

[56] Locke DE, Decker PA, Sloan JA (2007). Validation of single-item linear analog scale assessment of quality of life in neuro-oncology patients. J Pain Symptom Manag.

[57] Sloan JLC, Kuross S (1998). Randomized comparison of four tools measuring overall quality of life in patients with advanced cancer. J Clin Oncol.

[58] Higgins J, Se G (2011). Cochrane Handbook for Systematic Reviews of Interventions Version 5.1.0 [updated March 2011]. The Cochrane Collaboration Available from wwwhandbookcochraneorg.

[59] Toppinen-Tanner S, Ojajärvi A, Väänaänen A, Kalimo R, Jäppinen P (2005). Burnout as a predictor of medically certified sick-leave absences and their diagnosed causes. Behav Med.

[60] Letvak SA, Ruhm CJ, Gupta SN (2012). Nurses’ presenteeism and its effects on self-reported quality of care and costs. Am J Nurs.

[61] Pauly MV, Nicholson S, Polsky D, Berger ML, Sharda C (2008). Valuing reductions in on-the-job illness: 'Presenteeism' from managerial and economic perspectives. Health Econ.

[62] Burmeister EA, Kalisch BJ, Xie B (2019). Determinants of nurse absenteeism and intent to leave: an international study. J Nurs Manag.

[63] Budden JS, Moulton P, Harper KJ, Brunell M, Smiley R (2015). The 2015 National Nursing Workforce Survey. J Nurs Reg.

[64] The U.S. Nursing Workforce (2013). Trends in Supply and Education.

[65] Mbombi MO, Mothiba TM, Malema RN, Malatji M (2018). The effects of absenteeism on nurses remaining on duty at a tertiary hospital of Limpopo province. Curationis.

