# Supplementary material for: A cross-sectional study exploring the relationship between burnout, absenteeism, and job performance among American nurses
Source: BMC Nurs. 2019 Nov 21;18:57. doi: 10.1186/s12912-019-0382-7 (PMC6873742; doi:10.1186/s12912-019-0382-7)
Supplement: Supplementary file 1 — Additional file 1. Nurse survey. [file 12912_2019_382_MOESM1_ESM.doc]

APPENDIX 1: Nurse survey

1. What is your highest earned academic degree in or related to nursing?
   1. Licensed practical nurse
   2. Associate degree
   3. Baccalaureate degree in nursing
   4. Masters of science in nursing
   5. Doctorate of Nursing Practice (PhD)
   6. Other Doctorate of Nursing (PhD)
   7. Other: ____
2. How many years of experience do you have working in the field of nursing? ______ (enter #)
3. What is your current primary practice setting:
   1. Medical and surgical inpatient
   2. Intensive care
   3. Operating/recovery room
   4. Obstetrics
   5. Ambulatory/outpatient clinic
   6. Hospice
   7. Home health
   8. Non clinical, such as management
   9. Public health
   10. Other; _____________
4. Please mark the number (0-10) that best describing your feelings during the past week, including today.

Your level of fatigue, on average?

As Bad As As Good As

It Can Be It Can Be

0 1 2 3 4 5 6 7 8 9 10

1. What is your age: ___ (enter #)
2. What is your current relationship status:

a. Single

b. Married

c. Partnered

d. Widowed/widower

1. What is your gender: M F
2. Do you have any children?

a. yes

b. no

Maslach Burnout Inventory can be obtained here: <https://www.mindgarden.com/117-maslach-burnout-inventory>

World Health Organization Health and Work Performance Questionnaire can be obtained here: <https://www.hcp.med.harvard.edu/hpq/>
